# Nutritional Diversity in Native Germplasm of Maize Collected From Three Different Fragile Ecosystems of India

**DOI:** 10.3389/fnut.2022.812599

**Published:** 2022-04-11

**Authors:** Sapna Langyan, Rakesh Bhardwaj, Jyoti Kumari, Sherry Rachel Jacob, Ishwari Singh Bisht, Someswara Rao Pandravada, Archna Singh, Pratap Bhan Singh, Zahoor Ahmed Dar, Ashok Kumar, Jai Chand Rana

**Affiliations:** ^1^ICAR-National Bureau of Plant Genetic Resources (NBPGR), New Delhi, India; ^2^ICAR-National Bureau of Plant Genetic Resources (NBPGR), Hyderabad, India; ^3^Dryland Agriculture Research Station, SKUAST, Srinagar, India

**Keywords:** landraces, proximate composition, minerals, iron, antioxidants

## Abstract

Native germplasm resources are adapted to specific ecological niches. They have sustained over generations owing to the preference of local communities for their unique taste, the utility to particular dishes, and the low cost of cultivation. They may help eradicate malnutrition and act as a source for trait-linked genes. The present dataset comprises thirty-three native germplasm of maize collected from Rajasthan, Himachal Pradesh, and Andhra Pradesh states of India with an altitudinal variation of 386–2,028 m. They were evaluated for proximate composition, minerals, nutritional attributes, and antioxidant activity and compared with the standard values reported in the Indian Food Composition Table 2017 (IFCT2017). The nutritional profile showed moisture content in the range of 7.16–10.9%, ash 0.73–1.93%, crude protein 8.68–12.0%, crude fat 3.72–8.03%, dietary fiber 5.21–11.2%, and available carbohydrates 60.6–69.8%. Three accessions, namely, Malan 11 (7.06%), Malan 24 (7.20%), and Yellow Chamba Local 02 (8.03%) exhibited almost double the crude fat content as compared with the values notified in IFCT2017 (3.77). Total sugar content obtained was in the range of 5.00–11.3%, whereas the starch content was found between 50.9 and 64.9%. All the germplasm except Yellow Chamba Local reflected a higher protein content than reported values in IFCT2017 (8.80). Sathi, Safed Chamba Local, and Ragal Makka had nearly 12% protein content. Mineral malnutrition, mainly due to iron (Fe) deficiency, is a worldwide issue to science, humanity, and society. The mineral profile revealed that most germplasm had a higher iron content. Accessions with the iron content of nearly three times of IFCT2017 reported value were identified in germplasm belonging to three states. A negative relationship was observed between the altitude of the sample collection site and available carbohydrate content. In contrast, available carbohydrate showed inverse correlations with dietary fiber, protein, and fat content. The information generated in this study can be utilized to promote these germplasm as nutrifood, nutritional surveillance, labeling, and crop improvement programs.

## Introduction

India is known for its rich biodiversity of nutritious crops, such as cereals, millets, legumes, tubers, and medicinal plants. Germplasm are valuable genetic resources with high genetic variability and are well adapted to the local growing environment. Maize (*Zea mays* L.) is the most important food crop worldwide (1). Among all cereals, maize has the highest growth rate with maximum productivity. It is grown under diverse environmental conditions ranging from tropical to temperate regions. Maize is considered the most popular crop after wheat and rice, providing nutrients and primary raw material for the synthesizing significant biomolecules, such as oil, protein, starch, carlotenoids, food sweeteners, and biofuel (2–4). Maize is a relevant energy food source (5), so quantifying the nutritionally important grain constituents is vital for the best exploitation of the variability. Total phytate content and free phosphorous are essential parameters in food composition. They are the source of phosphorus in food and influence the bioavailability of several minerals and the digestibility of starch and protein (6, 7). Therefore, it is challenging to increase the amount of available P and reduce the amount of phytate in maize grain (8). Primary metabolites, such as carbohydrates, proteins, and lipids play crucial roles in the growth and development of animals and plants. Various epidemiological studies have shown that maize exhibits potential antioxidant activities (9), such as inhibition of colorectal carcinogenesis (10), anti-mutagenic (11), and radical scavenging activities (9). Physiological and morphological variation in maize is evident from its landraces studied by various researchers (12).

The landraces are still the staple diet for the local people. Hence, necessary for their nutritional security as these landraces are adapted to specific ecological niches. These landraces have sustained over generations due to local communities' appreciation for their unique taste, the utility to particular dishes, and the low cost of cultivation (12, 13). In this context, the maize germplasm represents a rich source of genetic variability and, therefore, may help identify the most suitable materials for developing nutritionally superior foods. Many studies have shown genetic and phenotypic variability for kernel composition in maize germplasm, but limited studies are available on the nutritional diversity within the germplasm (14–21). In this study, 33 maize germplasm accessions were collected from fragile ecosystems representing diverse agro-ecologies (namely, western Himalaya, hot arid, and southern plateau regions). Farmers prefer these landraces as commercial high-yielding varieties cannot sustain environmental pressures in those regions. They contribute majorly to staple food for the local community and are sold at a premium price because of their unique taste and perceived high nutritional value. Thus, information on the nutrient profile of these landraces is necessary for knowing their contribution to nutrient intake and consumer awareness through nutrition labeling.

## Materials and Methods

A representative set of 33 maize native germplasm was collected from agricultural fields after taking farmers' consent and documenting passport information from Adilabad, Udaipur, and Chamba districts of Telangana, Rajasthan, and Himachal Pradesh with an altitudinal variation of 386–2,028 m (Table 1). Five cobs of each accession were bulked to form one replicate; in total, two replicates were evaluated for biochemical attributes at ICAR-National Bureau of Plant Genetic Research (NBPGR), New Delhi, India. These regions are identified as fragile ecosystems by the Ministry of Environment, Forest & Climate Change, Government of India (22).

**Table 1:** Locational details of 33 maize germplasm used in study.

**Native germplasm collection no**.	**Accession no**.	**Source District**	**State**	**Latitude**	**Longitude**	**Altitude (m)**
Malan 05	IC0594423	Udaipur	Raj.	24° 25' 97	73° 20' 60	462
Malan 11	IC0273365	Udaipur	Raj.	25° 04'88.9	73° 33' 41.0	984
Malan 18	IC0594428	Udaipur	Raj.	24° 18' 791	73° 27' 931	415
Malan 21	IC0594420	Udaipur	Raj.	24° 25' 80	74° 06' 38	421
Malan 22	IC0594422	Udaipur	Raj.	24° 43' 01.5	73° 51'08.8	550
Malan 23	IC0280206	Udaipur	Raj.	24° 25' 97	73° 20' 60	462
Malan 24	IC0273363	Udaipur	Raj.	24° 17' 74.4	73° 34' 87	527
Malan 25	IC0280209	Udaipur	Raj.	24° 25' 39.9	74° 12' 35.7	415
Malan 36	IC0594438	Udaipur	Raj.	24° 25' 39.9	74° 12' 35.7	616
Sathi 01	IC0594420	Udaipur	Raj.	24° 28' 43.9	73° 45' 55.5	429
Sathi 04	IC0594429	Udaipur	Raj.	24° 41' 98.9	74° 02' 68.1	486
Sathi 09	IC0594430	Udaipur	Raj.	24° 28' 52.4	74° 19' 87.0	465
Sathi 11	IC0594424	Udaipur	Raj.	24° 23' 92.4	74° 06' 36.6	386
Sathi 12	IC0594421	Udaipur	Raj.	24° 28' 90.7	74° 12' 31.7	494
Sathi 13	IC0594425	Udaipur	Raj.	24° 28' 52.4	74° 19'87.0	465
Sathi 14	IC0594426	Udaipur	Raj.	24° 40' 48.7	74° 04' 26.8	495
Sathi 15	IC0280207	Udaipur	Raj.	24° 28' 45.3	74° 19' 59.7	472
Sathi 17	IC0594427	Udaipur	Raj.	24° 18' 83	73° 27' 950	624
Safed Chamba Local 01	IC0328979	Chamba	H.P.	32° 23' 04.2	76° 02' 27.0	1,290
Safed Chamba Local 02	IC0328957	Chamba	H.P.	32° 23' 7.8	76° 03' 30.7	1,365
Safed Chamba Local 03	IC0328844	Chamba	H.P.	32° 30' 0.3	76° 16' 10.8	1,410
Safed Chamba Local 04	IC0328849	Chamba	H.P.	32° 33' 35.3	76° 14' 56.2	2,028
Safed Chamba Local 05	IC0328888	Chamba	H.P.	32° 23' 02.1	76° 03' 28.4	1,428
Safed Chamba Local 07	IC0313197	Chamba	H.P.	32° 28' 54.7	76° 10' 51.8	1,228
Safed Chamba Local 08	IC0313200	Chamba	H.P.	32° 34' 41.2	76° 14' 23.0	1,453
Safed Chamba Local 09	IC0313227	Chamba	H.P.	32° 33' 38.7	76° 14' 49.2	2,020
Safed Chamba Local 10	IC0313203	Chamba	H.P.	32°49'55.3	76° 09'29.6	1,424
Yellow Chamba Local 01	IC0594406	Chamba	H.P.	32° 28' 7.4	76° 17' 0.8	1,760
Yellow Chamba Local 02	IC0594409	Chamba	H.P.	32° 27' 12.2	76° 15' 8.3	1,232
Red Chamba Local 01	IC0594399	Chamba	H.P.	32° 28' 7.4	76° 17' 0.8	1,760
Red Chamba Local 02	IC0594410	Chamba	H.P.	32° 29' 47.3	76° 21' 42.8	1,572
Chinna Makka	IC0627702	Telangana	A.P.	19° 28' 81	79° 06' 49	285
Ragal Makka	IC0623875	Telangana	A.P.	19° 11' 91	79° 13' 45	296

### Plant Material and Preliminary Screening of Maize Kernels

The maize kernels were taken from fully matured and sun-dried cobs, which were further oven-dried at 50°C for 24 h to reduce the moisture content and ground to a fine flour using a cyclotec mill with a 0.5 mm sieve. Flour samples were kept in air-tight sample containers and stored in desiccators to analyze various nutritional quality parameters.

### Proximate Composition Analysis

Samples were analyzed in duplicates following official and standard methods and the results are expressed as the mean. Proximate composition analysis was performed as follows: moisture content was obtained by drying 2 g of homogenized flour in the oven at 95°C for 2 h and repeat drying for 2 h until constant dry weight was obtained (AOAC 934.01), ash was obtained by incineration in a muffle furnace at 450°C (AOAC 938.08) for 5 h; crude protein by total nitrogen was obtained by the micro-Kjeldahl method (AOAC 2001.11) using the Foss Kjeltec nitrogen auto analyzer and Jone's factor 6.25; crude fat was extracted for 24 h in a soxhlet extractor (AOAC 920.39) using non-polar solvent petroleum ether (40–60°C). Total dietary fiber (DF) was obtained by enzymatic-gravimetric (AOAC 985.29) method using the Megazyme K-TDF kit (Megazyme, India). Available carbohydrates were calculated by the difference method according to the following equation:


$$\begin{array}{l} \left[100-\left(mositure+ash+C.\;protein+C.\;fat+DF\right)\right] \end{array}$$

### Estimation of Total Soluble Sugars, Total Phenols, Antioxidant Activity, Total Starch and Phytate, and Total Minerals

About 100 mg of sample was extracted three times in hot 80% ethanol and centrifuged at 10,000 g for 10 min, and supernatants were pooled, and volume was made up to 25 ml. Ethanolic extract was dried on a boiling water bath and, the residue was dissolved in 10 ml of distilled water. This extract was used for estimation of total soluble sugars (includes monosaccharide, disaccharides, and oligosaccharides), total phenols and total antioxidant activity as per the anthrone reagent method (23), Folin–Ciocalteu reagent method (24), and cupric ion reducing antioxidant capacity (25), respectively. The residue obtained after centrifugation from the ethanolic extraction step was used to estimate starch content as per AOAC 996.11 using the Megazyme K-TSTA kit (Megazyme, India). Total phytate and phosphorous were obtained using the Megazyme K-PHYT kit (Megazyme, India).

About 500 mg of homogenized samples were digested in 10 ml concentrated nitric acid and, volume was made up to 25 ml with double distilled water. Minerals {iron (Fe), copper (Cu), zinc (Zn), potassium (K), sodium (Na), and magnesium (Mg)} were estimated on an atomic absorption spectrophotometer (AAS220 Varian Fast Sequential, Australia) according to the method of AOAC 985.35. ASFRM-14 (Rice flour) and ASFRM-6 (fish meal) food reference materials obtained from the Institute of Nutrition, Mahidol University, Thailand, were used to validate the methods and ascertain recovery for proximate components and minerals.

### Data Analysis

All the samples were processed in duplicate, and a completely randomized design was used for analyzing results. The results were expressed in univariate and multivariate statistics. Two-tailed Pearson's correlations at a significance level of 1 and 5%, principal component analysis (PCA) based on Eigen values, and hierarchical cluster analysis (HCA) based on squared Euclidean distance using Ward's method were performed using the SPSS version 17 software.

## Results

### Nutritional Profile Variability, Proximate Composition Analysis, and Antioxidant Activity

The wet-laboratory experiments showed that maize grains contain moisture content in the range of 7.16–10.9%, ash (0.73–1.93%), crude protein (8.68–12.0%), crude fat (3.72–8.03%), dietary fiber (5.21–11.2%), and available carbohydrates by difference (60.6–69.8%) (Table 2A). Out of 33 accessions evaluated, 10 showed high protein content (11.7–12.0%) with the highest value of 12% protein in Safed Chamba Local 09 and Safed Chamba Local 05. Yellow Chamba Local 02 had 8% crude fat, whereas Red Chamba Local 02 showed the highest dietary fiber content. All but one, germplasm exhibited more protein content when compared with reported values in IFCT2017 (26) (8.80 ± 0.49). Three native germplasm, namely, Malan 11, Malan 24, and Yellow Chamba Local 02 have more than two times the fat content than IFCT2017 values. These germplasm show a widened base of genetic diversity based on proximate composition. Total soluble sugars obtained were in the range of 5.00–11.30%, and the starch content varied from 50.9 to 64.9%. The values obtained for total soluble sugar are more than five times compared with the IFCT2017 (1.66 ± 0.04%). IFCT2017 values for total sugars include only monosaccharides and disaccharides. In contrast, our method measures the mono, di, and oligosaccharides, which may have also contributed to our samples' showing higher amounts of total sugars. However, it is important to note that sum of total soluble sugar and total starch is in close agreement with the total available carbohydrate by difference. It is evident from the high correlation *R*^2^ = 0.911 (Table 3) and *p* of 0.526 (Table 4) from pair *T*-test signifying the differences between the two are non-significant. Total ash content ranged from 0.73 to 1.93%, with the highest ash content in Safed Chamba Local 10. Total phosphate was obtained in the range of 0.13–0.37%, whereas; phytate was in the range of 0.25–1.01% (Table 2B). Many of the problems associated with phosphorus in maize grain are not due to the concentration of the total phosphorus *per se*, but rather to the fact that most of it are bound to inositol in the form of phytate, which is a highly negatively charged and complexes cations with it and thus reduces the bioavailability of minerals. Total phenols are another group of compounds that bind minerals and reduce their bioavailability but are also preferred as antioxidants. Total phenols content ranged from 0.502 to 1.65% gallic acid equivalent (GAE). In addition, good variability was observed for cupric reducing antioxidant capacity in our samples, ranging from 1.19 to 2.98% GAE.

**Table 2A:** Variability in proximate composition, total soluble sugar and total starch content in maize germplasm.

**Native germplasm collection no**.	**Cluster No**.	**Altitude**	**Moisture (%)**	**Ash** **(%)**	**Protein (%)**	**CF** **(%)**	**DF** **(%)**	**Choavl (%)**	**Sugar (%)**	**Starch (%)**	**Sugar+starch (%)**
Ragal Makka	1	296	10.1	1.04	11.9	4.31	5.29	67.4	6.92	61.1	68.0
Sathi 11	1	386	7.16	1.54	11.7	6.88	6.74	66.0	7.09	58.9	66.0
Malan 18	1	415	8.21	0.968	9.83	4.82	7.06	69.1	7.09	61.6	68.7
Malan 25	1	415	8.58	1.70	10.3	4.30	8.38	66.7	5.62	60.2	65.8
Malan 21	1	421	8.63	1.33	10.9	3.78	5.59	69.8	6.12	64.9	71.0
Malan 23	1	462	10.9	0.904	10.2	4.30	7.19	66.5	6.76	58.9	65.7
Sathi 09	1	465	8.99	1.00	11.9	4.61	8.87	64.6	7.25	56.6	63.9
Sathi 15	1	472	7.65	0.881	11.7	3.94	9.52	66.4	7.25	58.5	65.8
Sathi 04	1	486	8.92	0.972	11.9	5.19	6.94	66.1	6.92	57.5	64.4
Sathi 01	1	494	9.30	1.14	11.7	4.95	6.52	66.4	8.07	57.1	65.2
Sathi 14	1	495	8.91	0.833	10.4	6.88	7.96	65.1	6.29	57.6	63.9
Malan 24	1	527	8.78	1.36	10.3	7.20	5.21	67.2	7.29	60.2	67.5
Malan 22	1	550	8.21	0.729	10.1	6.57	8.23	66.2	6.39	58.9	65.3
Sub-cluster 1 average			8.80	1.11	11.0	5.21	7.19	66.7	6.85	59.4	66.2
Malan 05	2	616	8.63	1.54	10.4	4.74	6.75	67.9	6.16	62.0	68.2
Sathi 12	2	624	10.4	1.49	9.14	4.24	6.33	66.4	6.09	60.7	66.8
Sub-cluster 2 average			9.52	1.52	9.77	4.49	6.54	67.2	6.13	61.4	67.5
Chinna Makka	3	285	7.72	1.53	11.1	4.30	6.06	69.3	8.25	62.6	70.9
Sathi 17	3	429	8.98	1.80	9.01	5.38	6.58	68.2	11.3	56.9	68.2
Malan 36	3	462	7.95	1.07	10.9	6.41	10.4	63.3	5.98	58.2	64.2
Sathi 13	3	465	8.87	1.17	10.7	6.57	6.06	66.6	6.79	58.9	65.7
Sub-cluster 3 average			8.38	1.39	10.4	5.67	7.28	66.9	8.08	59.2	67.2
Red Chamba Local 02	4	1,572	9.39	1.27	11.5	6.02	11.2	60.6	6.44	55.1	61.5
Red Chamba Local 01	4	1,760	9.99	1.37	9.89	5.07	8.77	64.9	5.63	59.2	64.8
Safed Chamba Local 02	4	1,760	9.22	1.30	11.4	6.78	9.36	61.9	6.93	54.1	61.0
Safed Chamba Local 03	4	2,020	8.48	1.16	11.7	5.89	8.74	64.0	6.30	60.0	66.3
Safed Chamba Local 04	4	2,028	9.43	1.26	11.2	5.33	6.94	65.9	5.00	58.9	63.9
Sub-cluster 4 average			9.30	1.27	11.1	5.82	9.00	63.5	6.06	57.5	63.5
Malan 11	5	984	8.99	1.13	10.6	7.06	6.15	66.0	5.96	59.1	65.1
Safed Chamba Local 08	5	1,228	10.3	1.10	9.96	5.26	9.78	63.6	10.8	52.1	62.9
Yellow Chamba Local 01	5	1,232	9.53	1.06	8.68	4.91	7.78	68.0	9.82	58.8	68.6
Safed Chamba Local 01	5	1,290	9.62	0.831	10.6	6.46	5.76	66.8	11.1	58.1	69.2
Yellow Chamba Local 02	5	1,365	10.7	1.47	9.40	8.03	5.29	65.1	8.21	57.0	65.2
Safed Chamba Local 09	5	1,410	10.1	1.86	12.0	5.79	8.94	61.3	8.05	53.9	62.0
Safed Chamba Local 10	5	1,424	9.89	1.93	11.8	5.79	8.38	62.2	7.77	53.9	61.7
Safed Chamba Local 05	5	1,428	9.33	1.16	12.0	6.76	6.41	64.3	6.63	56.4	63.0
Safed Chamba Local 07	5	1,453	9.83	1.00	9.27	3.72	8.44	67.7	9.03	58.2	67.2
Sub-cluster 5 average			9.81	1.28	10.5	5.98	7.44	65.0	8.60	56.4	65.0
Overall average			9.14	1.24	10.7	5.52	7.50	65.8	7.31	58.4	65.7
Standard deviation			0.89	0.31	0.98	1.15	1.57	2.25	1.57	2.69	2.56
Maize - Food Code A006 IFCT (26)			9.26	1.17	8.80	3.77	12.2	64.8	1.66	59.4	61.0

*(Samples are arranged as per cluster distribution in hierarchical clustering and ascending order of altitude). CF, Crude fat; DF, Dietary fiber*.

**Table 2B:** Variability in total phenols, anti-oxidant potential, phytate and minerals in maize germplasmvis-a-vis altitudinal differences.

**Native germplasm collection no**.	**Cluster no**.	**Altitude**	**Phenol % GAE**	**Cuprac % GAE**	**Phytate (%)**	**Total P (%)**	**Cu (μgg^**−1**^)**	**Zn** **(μgg^**−1**^)**	**Fe (μgg^**−1**^)**	**K** **(μgg^**−1**^)**	**Na (μgg^**−1**^)**	**Mg** **(μgg^**−1**^)**
Ragal Makka	1	296	0.504	1.42	0.417	0.127	0.409	16.7	54.9	3,858	165	1,544
Sathi 11	1	386	0.764	1.67	0.641	0.263	1.25	21.1	46.4	4,057	167	1,602
Malan 18	1	415	0.705	2.02	0.638	0.262	2.38	8.22	45.8	3,774	158	1,704
Malan 25	1	415	0.961	1.67	0.493	0.158	0.248	18.1	55.3	3,929	151	1,722
Malan 21	1	421	0.964	1.91	0.689	0.283	0.909	15.3	66.2	4,205	175	1,482
Malan 23	1	462	1.02	2.14	0.629	0.263	0.412	12.2	37.7	3,900	180	1,643
Sathi 09	1	465	0.919	1.67	0.643	0.261	8.92	11.3	28.5	3,616	142	1,491
Sathi 15	1	472	0.964	1.78	0.584	0.234	0.913	18.6	80.6	3,678	153	1,615
Sathi 04	1	486	1.26	1.87	0.559	0.233	1.97	18.8	52.2	3,689	173	1,497
Sathi 01	1	494	0.884	1.78	0.655	0.255	0.978	19.8	33.6	4,196	136	1,767
Sathi 14	1	495	1.11	1.54	0.655	0.265	1.54	15.2	40.5	3,904	144	1,684
Malan 24	1	527	0.976	1.42	0.463	0.202	1.55	12.5	61.5	3,785	177	1,341
Malan 22	1	550	0.514	1.67	0.632	0.264	2.13	10.6	43.5	4,196	131	1,326
Sub-cluster 1 average			0.888	1.74	0.592	0.236	1.82	15.3	49.7	3,907	158	1,571
Malan 05	2	616	0.499	1.43	0.933	0.339	2.62	21.6	78.2	4,218	212	2,666
Sathi 12	2	624	1.65	2.98	0.392	0.186	1.84	27.8	66.6	4,430	185	2,412
Sub-cluster 2 average			1.07	2.21	0.663	0.263	2.23	24.7	72.4	4,324	199	2,539
Chinna Makka	3	285	0.543	1.55	0.559	0.176	0.164	23.4	65.7	3,323	173	1,338
Sathi 17	3	429	1.17	2.02	0.452	0.206	2.32	29.1	37.8	3,410	157	1,352
Malan 36	3	462	0.668	1.19	0.824	0.318	2.31	9.60	48.6	2,347	167	1,281
Sathi 13	3	465	0.691	1.43	0.721	0.285	1.58	13.6	27.4	3,263	143	1,175
Sub-cluster 3 average			0.767	1.55	0.639	0.246	1.59	18.9	44.9	3,086	160	1,287
Red chamba local 02	4	1,572	0.429	1.66	0.783	0.312	1.08	14.2	70.0	3,695	166	2,634
Red chamba local 01	4	1,760	0.652	1.43	0.588	0.242	7.39	1.59	42.5	3,892	155	2,193
Safed chamba local 02	4	1,760	0.779	1.19	0.634	0.251	2.91	7.86	73.4	4,192	185	2,559
Safed chamba local 03	4	2,020	0.714	1.67	0.765	0.298	1.79	5.77	46.9	4,590	133	2,643
Safed chamba local 04	4	2,028	0.735	1.66	0.774	0.304	1.79	4.97	53.5	4,570	138	2,848
Sub-cluster 4 average			0.662	1.52	0.709	0.281	2.99	6.88	57.3	4,188	155	2,575
Malan 11	5	984	0.422	1.19	0.577	0.255	4.98	6.34	59.7	3,198	90.1	2,278
Safed chamba local 08	5	1,228	0.792	2.25	0.712	0.297	4.58	28.2	60.9	3,688	167	1,375
Yellow chamba local 01	5	1,232	0.578	1.55	0.541	0.219	2.12	3.37	50.6	3,588	152	1,479
Safed chamba local 01	5	1,290	0.926	2.02	0.695	0.285	1.07	19.6	33.9	3,906	158	1,830
Yellow chamba local 02	5	1,365	0.556	1.66	0.473	0.254	1.15	2.36	33.8	2,971	145	1,209
Safed chamba local 09	5	1,410	0.807	1.67	1.01	0.366	1.22	56.8	25.8	3,666	143	2,059
Safed chamba local 10	5	1,424	0.618	1.66	0.899	0.339	1.29	76.8	39.2	3,268	139	2,138
Safed chamba local 05	5	1,428	0.625	1.78	0.471	0.215	2.64	4.77	45.2	3,388	144	2,056
Safed chamba local 07	5	1,453	0.691	1.79	0.247	0.184	1.91	15.4	26.3	3,340	130	1,430
Sub-cluster 5 average			0.668	1.73	0.625	0.268	2.33	23.7	41.7	3,446	141	1,762
Overall average			0.790	1.71	0.629	0.255	2.13	17.3	49.5	3,749	156	1,799
Standard deviation			0.26	0.34	0.16	0.05	1.88	15.0	15.3	471	22	487
Maize food code A006 IFCT (26)			0.032		0.646	0.279	4.5	22.7	24.9	2,910	44.4	1,450

*(Samples are arranged as per cluster distribution in hierarchical clustering and ascending order of altitude)*.

**Table 3:** Pearson correlation between different nutrient components and altitude in native maize germplasm.

	**Moisture**	**Ash**	**Protein**	**Crude** **fat**	**Dietary** **fiber**	**Available** **carbohydrate**	**Sugar**	**Starch**	**Sugar** **+Starch**	**Phenol**	**Cuprac**	**Phytate**	**Total P**	**Cu**	**Zn**	**Fe**	**K**	**Na**	**Mg**
Altitude	0.415^*^	0.117	0.046	0.260	0.336	−0.559^**^	0.022	−0.448^**^	−0.461^**^	−0.316	−0.117	0.206	0.346^*^	0.194	−0.046	−0.079	0.145	−0.275	0.612^**^
Moisture		0.074	−0.266	−0.043	−0.069	−0.265	0.221	−0.382^*^	−0.268	0.107	0.334	−0.119	0.006	0.089	0.151	−0.300	0.005	−0.022	0.149
Ash			0.019	0.019	−0.076	−0.161	0.031	−0.092	−0.077	0.024	−0.001	0.201	0.104	−0.133	0.582^**^	0.054	−0.056	0.169	0.224
Protein				0.076	0.153	−0.429^*^	−0.322	−0.194	−0.399^*^	−0.152	−0.306	0.434^*^	0.268	−0.059	0.236	0.069	0.095	−0.067	0.257
Crude fat					−0.083	−0.440^*^	−0.014	−0.376^*^	−0.404^*^	−0.260	−0.397^*^	0.171	0.305	0.040	−0.132	−0.195	−0.254	−0.260	0.017
Dietary fiber						−0.667^**^	−0.074	−0.533^**^	−0.606^**^	−0.134	−0.124	0.367^*^	0.374^*^	0.270	0.109	0.099	−0.101	−0.050	0.226
Available carb							0.127	0.792^**^	0.911^**^	0.161	0.188	−0.471^**^	−0.515^**^	−0.202	−0.271	0.083	0.128	0.147	−0.408^*^
Sugar								−0.373^*^	0.221	0.075	0.276	−0.162	−0.075	−0.048	0.280	−0.289	−0.257	0.000	−0.419^*^
Starch									0.822^**^	0.041	0.010	−0.244	−0.359^*^	−0.227	−0.363^*^	0.279	0.252	0.222	−0.088
Sugar+starch										0.090	0.180	−0.351^*^	−0.420^*^	−0.267	−0.206	0.116	0.107	0.232	−0.351^*^
Phenol											0.667^**^	−0.256	−0.222	−0.079	0.163	−0.008	0.295	0.294	−0.096
Cuprac												−0.233	−0.127	−0.130	0.227	−0.033	0.316	0.224	−0.019
Phytate													0.910^**^	0.016	0.396^*^	0.014	0.080	0.079	0.376^*^
Total P														0.116	0.307	−0.121	0.004	−0.036	0.342
Cu															−0.231	−0.124	−0.083	−0.201	0.101
Zn																−0.126	−0.090	0.054	0.041
Fe																	0.265	0.498^**^	0.348^*^
K																		0.218	0.519^**^
Na																			0.032

* *, Correlation is significant at the 0.05 level (2–tailed)*.

** *, Correlation is significant at the 0.01 level (2–tailed)*.

**Table 4:** Paired sample *T*-Test for testing significant differences between available carbohydrate and sum of sugar and starch.

**Pair**					***t***	**df**	**Sig. (2-tailed)**
	**Std. deviation**	**Std. error mean**	**95% Confidence interval of the difference**				
			**Lower**	**Upper**			
Available carbohydrates : sugar + starch	1.0596	0.1845	−0.2575	0.4939	0.641	32	0.526

*Standard Deviation, standard error of mean, degree of freedom, t-score, significance, diversity pair*.

### Estimation of Minerals

Minerals often serve as an important co-factor of enzymes and are often involved in biological reactions (27). Iron is an essential mineral for humans. The iron content ranged from 25.8 to 80.6 μg/g. Copper is a co-factor of many antioxidants (28), and it ranged from 0.16 to 8.92 μg/g with the highest content exhibited by Sathi 09 landrace. Zinc ranged from 1.59 to 76.8 μg/g, potassium (2,347–4,590 μg/g), sodium (90.1–212 μg/g), and magnesium (1,174–2,848 μg/g). Potassium, magnesium, and sodium were high, being the macro-minerals for plants (Table 2B). Here, the mineral profile revealed that most of the landraces had a higher iron, potassium, sodium, and magnesium content than IFCT2017 values. Sathi 15 showed maximum iron content of 80.1 μg/gm and almost similar zinc content compared with IFCT2017 reported values.

### Correlation

Correlation coefficients among various quality attributes are given in Table 3. We found that the altitudinal differences and variability in native germplasm resulted in a highly significant negative correlation with available carbohydrate (−0.559), significant negative correlation with starch (−0.448), a highly significant positive correlation with magnesium, and a significant positive correlation with total phosphorus. Starch is a principal constituent of available carbohydrates; hence highly significant positive correlation (0.792) was observed between them. Further, it is observed that available carbohydrates are also showing a highly significant negative correlation with dietary fiber (−0.667) and a significant negative correlation with protein (−0.429) and crude fat (−0.440).

On the other hand, starch was negatively correlated with dietary fiber (−0.533) and sugar (−0.373) which means sugars and starch act as either diluent to other components or replace them in the endosperm. Most phosphorus is stored as phytate; hence a robust positive correlation (0.910) is observed between the two traits. Phytate showed a highly significant negative correlation (−0.47) with available carbohydrates and significant positive correlations with dietary fiber and protein. It implies that in breeding for developing high protein maize, there is a possibility of coinheritance for high phytate, dietary fiber, crude fat, and low available carbohydrate. Cupric reducing antioxidant capacity (CUPRAC) antioxidant activity assay showed a highly significant positive correlation with total phenols (0.667) and a negative correlation with crude fat (−0.397). It implies that phenols are the major antioxidant compounds present in maize kernels. Phytate and phenols are considered as the major anti-nutritional factors as they form a complex with minerals and reduce their bioavailability. However, we got a significant positive correlation of phytate with zinc (0.396) and magnesium (0.376) and no correlation between phenols and other minerals. There were no significant correlations of iron with phytate and phenol. So, there is a possibility of identifying accession with high iron, having low phytate and phenols content. Our study, could identify landraces, namely, Sathi 15 and Safed Chamba Local 02 with, an iron content of more than 70 μg/g and a total phytate concentration of less than 0.65%. At the same time, Malan 05 possesses 78.2 ug/g iron with phenols less than 0.5%.

### PCA and HCA

**Figure 1:**
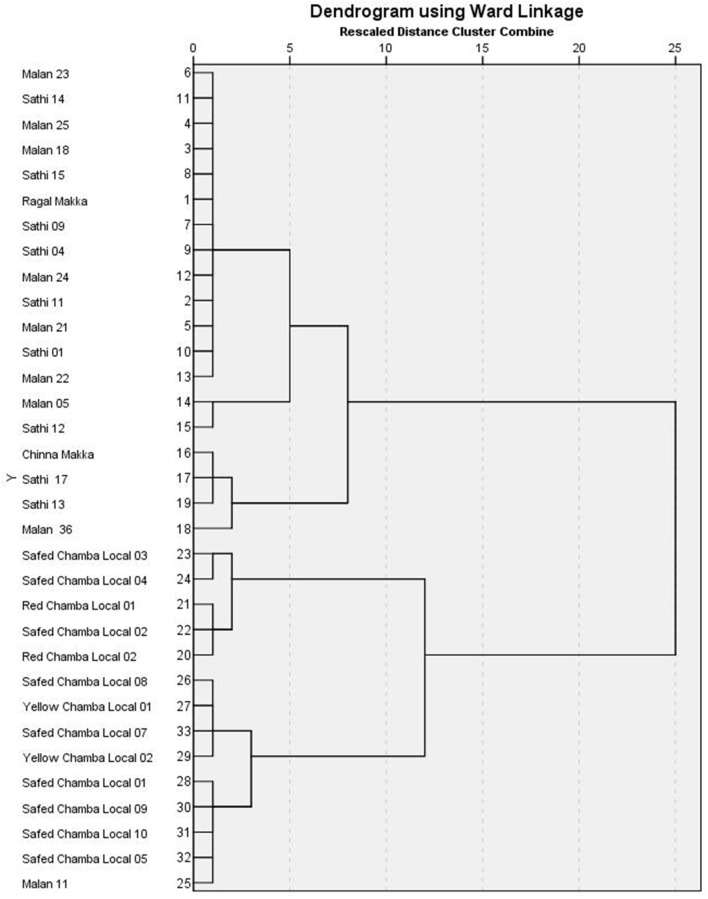
Scree plot for principal component analysis (PCA) in native maize germplasm.

**Figure 2:**
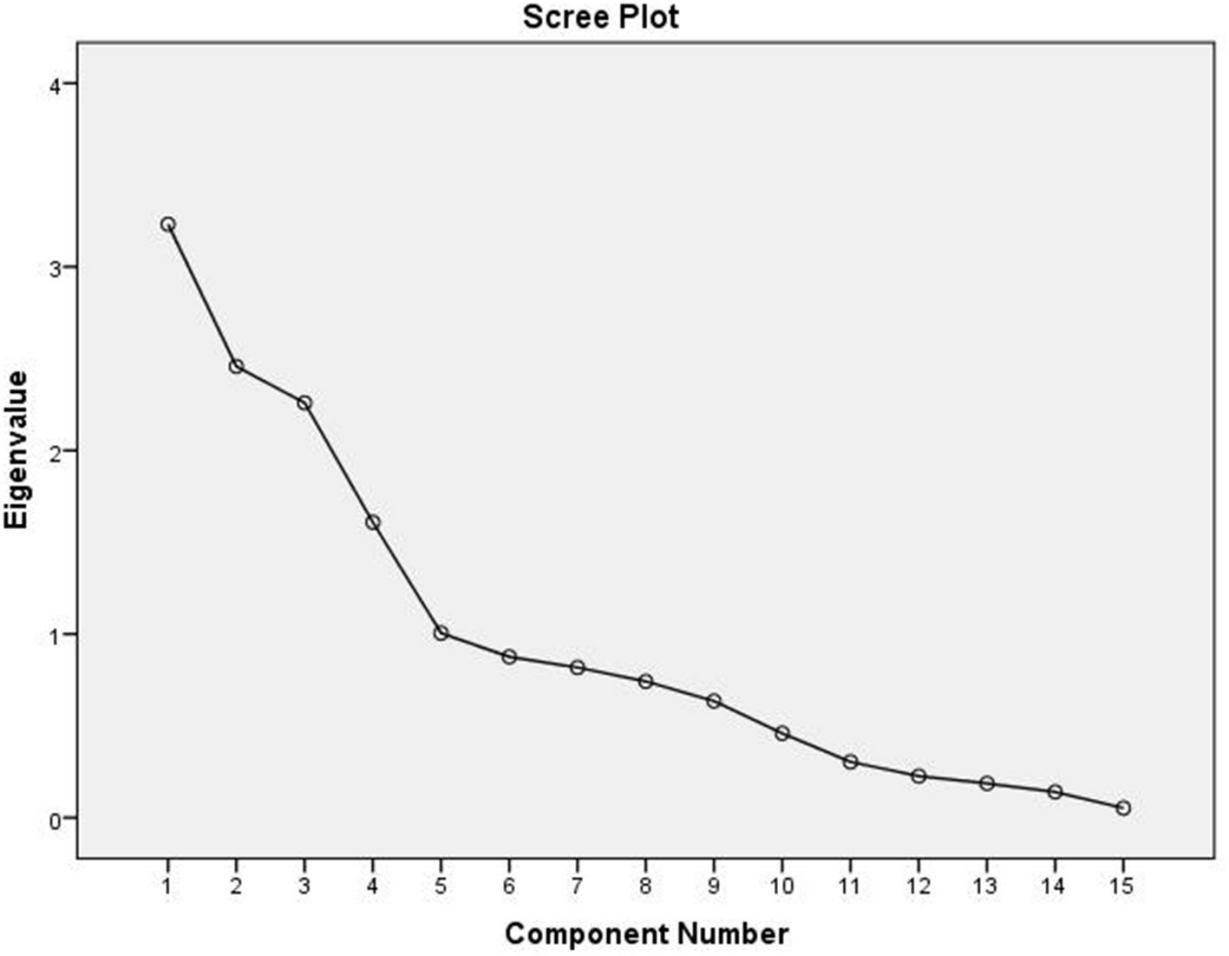
Hirerchial clustering based of native maize germplasm using wards' method.

The correlation between the traits indicates the possibility of finding a common axis to relate them and identify principal components contributing to variability. Kaiser-Meyer-Olkin (KMO) test (Table 5) was performed to examine the existence of a partial correlation between variables, and Bartlett's test of sphericity for verifying variables are related. KMO value of >0.5 and Bartlett's test significance <0.05 are essential for factor analysis. Accordingly, unrelated components, namely, TDF and Fe were removed as they decreased KMO value to less than 0.5. At the same time, total P and sugar + starch were not considered as their contribution is represented by phytate and available P, respectively. We obtained a KMO value of 0.537 and Bartlett's test significance <0.001, thus selected components to have a good relationship for the factor analysis. PCA revealed first five PCs have eigen values >1 (Tables 6, 7), explaining more than 70% variance, of which the first three PCs contributed to 53% variance (Table 8, Figure 1). Available carbohydrate, starch, phytate, protein, and crude fat content are the major contributors in the first PC for variability in maize accessions, followed by Zn, Cuprac, phenol, and moisture content in PC2. Sugar, K, and Mg significantly contribute to PC3, whereas Na, Cu, and ash have minimal contribution to variability. Thus, it is possible to group landrace accessions based on traits having a high contribution to variability and understand their relationship. Hierarchical clustering was done using the Wards' method and squared Euclidean distance (Figure 2). The HCA was preferred due to the high heterogeneity within groups. A total of two major clusters were found; cluster I has 19 accessions while cluster II has 14 accessions. Except for Malan 11, all accessions collected from Rajasthan and Telangana were in major cluster I, while accessions from Himachal Pradesh and Malan 11 were in major cluster II. Five sub-clusters were formed at a distance of 5, having 13, 2, 4, 5, and 9 accessions, respectively. Sub-cluster 1 had average values for most of the traits. At the same time Sub-cluster 2 having two members (namely, Malan 05 and Sathi 12) are unique, as both members have high available carbohydrate, total ash, zinc, iron, copper, sodium, potassium, magnesium, and low protein, crude fat, and dietary fiber. As Malan has white kernels and Sathi has yellow kernels, these two accessions can be used as parents for increasing the mineral density of Malan and Sathi maize, respectively, without affecting the kernel color. Sub-cluster 3 was similar to cluster I as most of the traits are in the average range except moisture and magnesium contents were the lowest. Sub-cluster 4 is characterized by the high content of protein, dietary fiber, magnesium, and low available carbohydrates. Sub-cluster 5 has high moisture and total sugars along with low starch and low sodium. Malan 11 and Malan 23 are the farthest apart, having significant differences in ash, crude fat, total phenols, copper, zinc, iron, sodium, and magnesium content. This variability can be exploited in crop improvement programs, particularly for minerals, phenol content, and hybrid vigor benefits.

**Table 5:** KMO and Bartlett's Test to test partial correlation and unrelated variables.

**Kaiser-Meyer-Olkin Measure of Sampling Adequacy**.		**0.537**
Bartlett's Test of Sphericity	Approx. Chi-Square	203.561
	Df	105
	Sig.	0.000

**Table 6:** Eigen values explaining component contribution in total variance.

**Component**	**Initial eigenvalues**		
	**Total**	**% of Variance**	**Cumulative %**
1	3.232	21.544	21.544
2	2.458	16.385	37.929
3	2.259	15.062	52.991
4	1.609	10.724	63.715
5	1.005	6.700	70.415
6	0.876	5.839	76.254
7	0.818	5.452	81.705
8	0.742	4.946	86.652
9	0.635	4.232	90.884
10	0.459	3.063	93.947
11	0.304	2.024	95.971
12	0.226	1.508	97.480
13	0.186	1.242	98.722
14	0.140	0.932	99.653
15	0.052	0.347	100.000

*Extraction method: principal component analysis*.

**Table 7:** Principal component matrix arranged with decreasing order of loading values.

	**Component**				
	**1**	**2**	**3**	**4**	**5**
Available carbohydrates	0.860	−0.257	0.043	−0.250	0.111
Starch	0.661	−0.415	0.462	−0.221	0.205
Phytate	−0.648	0.152	0.368	−0.214	−0.062
Protein	−0.562	−0.055	0.422	−0.182	−0.507
Crude fat	−0.549	−0.256	−0.290	0.042	0.017
Zn	−0.300	0.722	−0.013	−0.476	−0.039
Cuprac	0.519	0.681	0.001	0.243	−0.182
Phenol	0.478	0.546	0.101	0.167	−0.366
Moisture	−0.052	0.517	−0.309	0.483	0.323
K	0.219	0.207	0.703	0.372	−0.064
Sugar	0.159	0.383	−0.680	−0.177	−0.075
Mg	−0.403	0.183	0.635	0.403	0.295
Na	0.305	0.315	0.360	−0.205	0.076
Cu	−0.206	−0.175	−0.181	0.551	0.086
Ash	−0.240	0.475	0.131	−0.438	0.553

*Extraction method: principal component analysis*.

## Discussion

Proximate composition defines the primary nutrients of food; variability in them affects carbohydrate, protein, fat content, and the total calorific value of food (29). In this study, the estimate of total sugars in all, while the protein in all but one is higher than IFCT2017 values. Ullah et al. (30) reported a similar trend for different nutrients in maize grain, namely, moisture (9.20–10.9%), ash (0.70–1.30%), protein (7.71–14.6), fat (3.21–7.71%), crude fiber (0.80–2.32%), and carbohydrates (69.6–74.54%). Whereas 27 reported a range of major nutrients in Indian maize comprising protein (8–13%), starch (68–72%), oil (2–5%), and sugar (2–4%). The grains with a higher ash proportion contain a more significant portion of non-endosperm material (30) and provide a higher amount of different minerals. In a similar study, Aisha and El-Tinay (31) obtained ash in the range of 1.0–2.0%. Total ash content (Table 2A, average value 1.24% ± 0.34) was in close agreement with IFCT2017 reported value (1.17% ± 0.16). Andjelkovic et al. (32) reported the genetic variability in maize landraces' protein, oil, and starch content. The protein content varied from 10.1 to 14.8%, starch (66.4–71.5%), while oil content varied from 3.63 to 4.80%. They concluded that landraces showed exceptional nutritional quality diversity and are suitable for the future breeding program. In total 30 reported high oil (5.8–7.9%) and protein (10.6–12.4%) in the drought-tolerant mini core collection from Maize Research Institute genebank Zemun Polje, Serbia. Further, 31 showed very high variability in protein concentration that varied from 8.83 to 15.5% and starch concentration (67.4–75.3%). Their results are in close agreement with our findings as the native germplasm collected in our study belongs to fragile ecosystems where water stress is the most common occurrence.

In a similar study, Kumar et al. (20) at ICAR-NBPGR studied 51 accessions of maize germplasm from the northwest Himalayan region of India for agro-morphological traits and some of the quality traits. They found that the protein ranged from 9.89 to 13.3% and sugar content from 3.35 to 4.53%. They found that Jammu and Kashmir accessions had higher average protein, oil, sugar, and starch content values. In contrast, the accessions of Himachal Pradesh had more tryptophan and specific gravity. Then, they concluded that the set of maize germplasm was diverse and had traits for climate change and nutritional security and emphasized the need for more studies on the nutritional aspect of the maize germplasm.

Phytate act as a phosphorus reserve for different grain wherein nearly 90% phosphorus of maize kernel is found in the form of phytate (33). Phytate is inositol hexakis phosphate, a highly negatively charged compound, which strongly binds cations, such as Na, K, Ca, Mg, Cu, Zn, and Fe and reduces their bioavailability. At the same time, phytate helps prevent kidney stone formation, lowers blood glucose and lipids, and provides antioxidative and anticarcinogenic activities (34). The average phytate content (0.628 ± 0.16 %) reported in the present study is similar to the value reported in IFCT2017 (0.646 ± 0.19). Genetic variability in phytate contents of 54 landraces was observed, with values ranging from 0.114 to 0.413 and an average of 0.291% (7). Twenty-six populations had high, twenty intermediate, and eight low phytate content (7).

We found that most of our landraces estimated a higher mineral content than IFCT2017. A negative correlation between yield and minerals accumulation is well reported (35). Land races used in this study are collected from their native environments where the use of chemical fertilizers is either non-existent or minimal. These land races have comparatively less yield but are adapted to grow under natural farming conditions and are preferred for low input requirements. An earlier study by Kravić et al. (27) investigated thirteen maize local landraces for Fe, Mn, Zn, β-carotene, and phytate content in grain. They found that genotype LL3 exhibited the highest Fe content and the highest β-carotene content (25.63 μg g^−1^) and the lowest phytate/β-carotene ratio, which could be considered potential sources of favorable genes for breeding programs to improve the nutritional quality. Jaradat and Goldstein (36) studied the ionome of maize kernels from 13 breeding populations of high protein lines comprising 1,348 accessions. They obtained good variability for different minerals as depicted by average value ± SDs and ranges in mg/Kg for Mg, K, P, Cu, Fe, and Zn- 1,397 ± 145, 1,027–1,825; 3,626 ± 433, 2,332–5,166; 0.345 ± 0.035, 0.256–0.451; 2.7 ± 0.98, 0.92–8.7; 23.6 ± 4.35, 13–54.7; 23.9 ± 3.35, 15.1–41.9, respectively. Their findings were in close agreement with our results, except for iron, where we have found relatively higher values. Various approaches to the high phytate problems include engineering crops to express high levels of phytase enzyme in seeds (37), breeding for low-phytate maize, or through the recurrent selection that uses the indigenous quantitative genetic variation (38). Our study could identify the germplasm with low phytate content (<0.4%), namely, Safed Chamba Local 07 and Sathi 12.

Phenolic compounds are secondary metabolites that play an essential role in plant defense to various stresses, providing specific flavor, color, fragrance, and medicinal value. Several reports are also available on their anti-cancer, anti-aging, and anti-diabetic activities (39–41). Total phenol estimation is an important indicator of the accumulation of polyphenols, and the total content in our samples ranged from 0.422 to 1.65% GAE. However, phenols are implicated in reducing the bioavailability of minerals by complexing them, though they play a positive role through the strong antioxidant activity and reduction in food's glycemic load by decreasing the activity of amylases. Antioxidant activity is an important measure of capacity to neutralize free radicals and other oxidizing species. Several methods are used and reported for the purpose, such as **2,2'-azino-bis(3-ethylbenzothiazoline-6-sulfonic acid** (ABTS) assay, **2,2-diphenyl-1-picrylhydrazyl** (DPPH) assay, ferric reducing antioxidant power (FRAP) assay, and oxygen radical absorbance capacity (ORAC) assay. However, we chose the CUPRAC assay as this method measures cupric reducing capacity in neutral pH. It shows reactivity with most flavonoids and no reaction with reducing sugars and organic acids (25).

Environment plays a vital role in phenotypic expression; hence its effect on nutrient composition merits investigation. Our study reports the negative relationship of altitude with available carbohydrate and starch. It agrees with Xue-jun et al. (42) report, which found maize samples collected from high altitudes had low starch, but no correlation was observed with protein. The negative correlation of protein with starch could be due to their spatial presence in the endosperm, and an increase in the quantity of one will decrease the other. Our results agree with the findings of other researchers (43–46). A positive protein correlation with oil might have originated from the relative weight distribution of endosperm and germ in the mature maize kernel. These results are in close agreement with Panthee et al. (47). Okporie and Oselebe (48, 49) reported that there would be no severe barrier in selecting for both the high protein and high oil in maize.

The multivariate analysis, particularly PCA and HCA, are used to reduce the dimensions, identify traits that have maximum contribution in variability, and understand the relationships between germplasm lines (50, 51). Several workers have reported PC1 has 1.3-fold higher Eigen values than that of PC2, which is commensurate with our findings of Eigen values of 3.232 (PC1) and 2.458 (PC2) (51–53). Sample size, number of variables, degree of component identification, component saturation, partial relationship between traits, and extent of variability contribute to retained components. In chemometrics, mainly 2–4 components are practically significant, and scree generally provides the most accurate rule (54, 55). Scree plot (Figure 2) shows major inflection at component 4; thus first 3 PCs are sufficient to explain the significant variance in data. First, PC explains maximum variance; in our results, the major contributors are available carbohydrate, starch, phytate, protein, and crude fat content. Thirty one has also reported that starch and protein have maximum influence on variability in the kernels of maize inbred lines. Carbohydrate, protein, and oil are majorly influenced by genotype and environment. Landraces collected from diverse and distant agro-ecologies are distinct genetically and carry the influence of the environment (56), which has contributed to the high variability and contributing components. HCA is used to group similar objects in one cluster, grouping accessions from Rajasthan and Telangana in cluster I; and accessions from Himachal Pradesh and lone accession from Rajasthan Malan 11 in cluster II indicates the influence of altitude on adaptability and composition. Xue-jun et al. (42) have also reported the influence of altitude on the composition in accessions collected from different altitudes of Gansu and Yunnan province, China.

## Conclusion

Native germplasm maintained by farmers has evolved over the generations through a complex adaptation process of different original genotypes to diverse climatic and soil conditions; farmer's choice and selection criteria are the most accessible part of maize biodiversity. Genetic diversity of maize germplasm for grain quality characterization aids efficient exploring of the allelic variation for genetic improvement of economically desirable traits, such as grain quality. The germplasm collections are the source of potentially valuable traits and alleles to improve modern varieties. It could be concluded that much of the tested germplasm show an exceptional kernel quality, a wide genetic diversity based on the proximate composition, minerals, phytate, phenols, and antioxidants. It makes them suitable for use as the reference set, source of favorable traits and future crop improvement programs. A total number of six native germplasm, namely, Ragal Makka, Sathi 15, Malan 05, Red Chamba Local 02, Safed Chamba Local 02, and Safed Chamba Local 10 are selected for use in crop improvement programs aimed to broaden the genetic base and improve the nutritional composition, particularly for protein, iron, and zinc.

## Data Availability Statement

The original contributions presented in the study are included in the article/supplementary material, further inquiries can be directed to the corresponding author/s.

## Author Contributions

ISB conceived the idea. JCR, SRP, AS, and PBS collected the landraces. RB and SL did the nutritional analysis and wrote the manuscript. JCR, JK, and SRJ edited the manuscript. ZAD and AK did the formal analysis. All authors contributed to the article and approved the submitted version.

## Funding

We are thankful to Director ICAR-NBPGR, New Delhi, for institutional support. This work was supported by externally funded projects, namely, the World Bank funded project through the National Agricultural Innovation Project (NAIP) scheme and the Global Environment Facility (GEF) of the United Nations Environment Program (UNEP) project on Mainstreaming agricultural biodiversity conservation and utilization in the agricultural sector to ensure ecosystem services and reduce vulnerability.

## Conflict of Interest

The authors declare that the research was conducted in the absence of any commercial or financial relationships that could be construed as a potential conflict of interest.

## Publisher's Note

All claims expressed in this article are solely those of the authors and do not necessarily represent those of their affiliated organizations, or those of the publisher, the editors and the reviewers. Any product that may be evaluated in this article, or claim that may be made by its manufacturer, is not guaranteed or endorsed by the publisher.
